# Complement After Trauma: Suturing Innate and Adaptive Immunity

**DOI:** 10.3389/fimmu.2018.02050

**Published:** 2018-09-24

**Authors:** Shinjini Chakraborty, Ebru Karasu, Markus Huber-Lang

**Affiliations:** Institute of Clinical and Experimental Trauma-Immunology, University Hospital of Ulm, Ulm, Germany

**Keywords:** complement, trauma, innate immunity, adaptive immunity, T-cells, B-cells

## Abstract

The overpowering effect of trauma on the immune system is undisputed. Severe trauma is characterized by systemic cytokine generation, activation and dysregulation of systemic inflammatory response complementopathy and coagulopathy, has been immensely instrumental in understanding the underlying mechanisms of the innate immune system during systemic inflammation. The compartmentalized functions of the innate and adaptive immune systems are being gradually recognized as an overlapping, interactive and dynamic system of responsive elements. Nonetheless the current knowledge of the complement cascade and its interaction with adaptive immune response mediators and cells, including T- and B-cells, is limited. In this review, we discuss what is known about the bridging effects of the complement system on the adaptive immune system and which unexplored areas could be crucial in understanding how the complement and adaptive immune systems interact following trauma.

## Complement activation drives innate immunity early after polytrauma

Well-known ancient physician and philosopher Hippocrates from Kos (ca. 460–370 B.C.) had defined some interventions in trauma care, which had been radical at that time, eventually, some of them proving to be clinically relevant (1). Hippocratic Medicine followed the humoral pathology and defined the “four humors” or fluids of the body (among them blood), which had to be rebalanced to achieve healing. Almost as a foresight of the complement response after trauma, Hippocrates clearly pointed out the importance of humoral components in the pathophysiological course of diseases, thus being the first to indicate the involvement of the fluid phase and its potential relevance. Drawing on this age-old concept, complement activation was formerly defined as “disseminated intravascular multiple systems activation” following trauma as in a major burn injury (2). The cellular immune system with “pre-programmed responses” had been proposed to be of less importance after severe tissue injury compared to the mediator systems of complement components and other opsonins (3). Supporting this observation, activation of the humoral systems, including the coagulation and complement systems, was described to occur early after multiple injury because complement activation products, the anaphylatoxins C4a and C3a, were elevated and associated with injury severity and infectious complications (4, 5). These preliminary observations stimulated a growing interest in trauma-associated complement activation, which extended from temporal to spatial activation tendencies. Two studies are of relevance in this regard. In one clinical study, early after trauma (< 48 h) including multiple fractures, blunt abdominal trauma and blunt chest trauma with rib fractures, high C3a/C3 ratio was useful to segregate adult respiratory distress syndrome (ARDS) from non-ARDS patients (6). In a later study, plasma samples from patients were obtained as early as 30 min post-trauma comprising of penetrating injury or severe head injury and with a mean injury severity score (ISS) of 17, without any resuscitative fluid replacement therapy. Here it was found that the soluble terminal complement complex (TCC) sC5b-9 and Bb (activated factor B) were associated with high ISS (7). On comparing the two studies, several similarities and discrepancies can be summarized. Both studies identified an early activation of the alternative pathway, however, opposing in whether classical complement activation preceded or followed alternative pathway activation. Interesting to note in here is the difference of time-points in the two studies (30 min vs. < 48 h post-trauma) and the fluid resuscitation that was received by patients in the former study as opposed to none received when blood samples were collected for the latter. Apart from this temporal association, the site and pattern of injury could also modulate activated complement factors and complexes differentially, as was described in a small cohort study proposing that the appearance of the TCC and C3dg as early as 24 h after trauma was mainly seen in patients who had suffered a thorax trauma (8).

When complement activation products were individually implicated in trauma-induced local and systemic trauma complications, several groups found that early C3a generation and depletion of C3 with a resultant increase in the C3a/C3 ratio were associated with the development of acute lung injury, ARDS, sepsis, multiple organ dysfunction and consequently a poor prognosis (6, 9–12). Likewise, anaphylatoxin C5a appears in the circulation of humans within 20 min post polytrauma and its levels have been related to the mortality rate (10). Exposure to C5a may result in significantly delayed neutrophil apoptosis early after trauma, thereby causing enhanced host damage through the recruitment and accumulation of neutrophils at the injury site (13). The underlying causes of rapid local and systemic complement activation via all pathways may be manifold and some remain speculative, for example, tissue hypoperfusion with acidic (micro)environments, natural antibodies, reactive oxygen species, coagulation-complement crosstalk, release of non-specific proteases with subsequent cleavage of complement components, exposure to pathogen-associated molecular patterns (PAMPs) in a synchronic shock situation, artificial surfaces like catheter materials and transfusion of blood products, including complement components in fresh frozen plasma among others (14–18). Uncontrolled complement activity can be further augmented by dysregulated levels of complement regulators, including C4b-binding protein and factor I (10). Additionally, an early depletion of central complement components causing trauma-induced complementopathy with declining complement hemolytic activity and manifestation of signs of immunodeficiency have been reported (10, 19).

**Figure 1:**
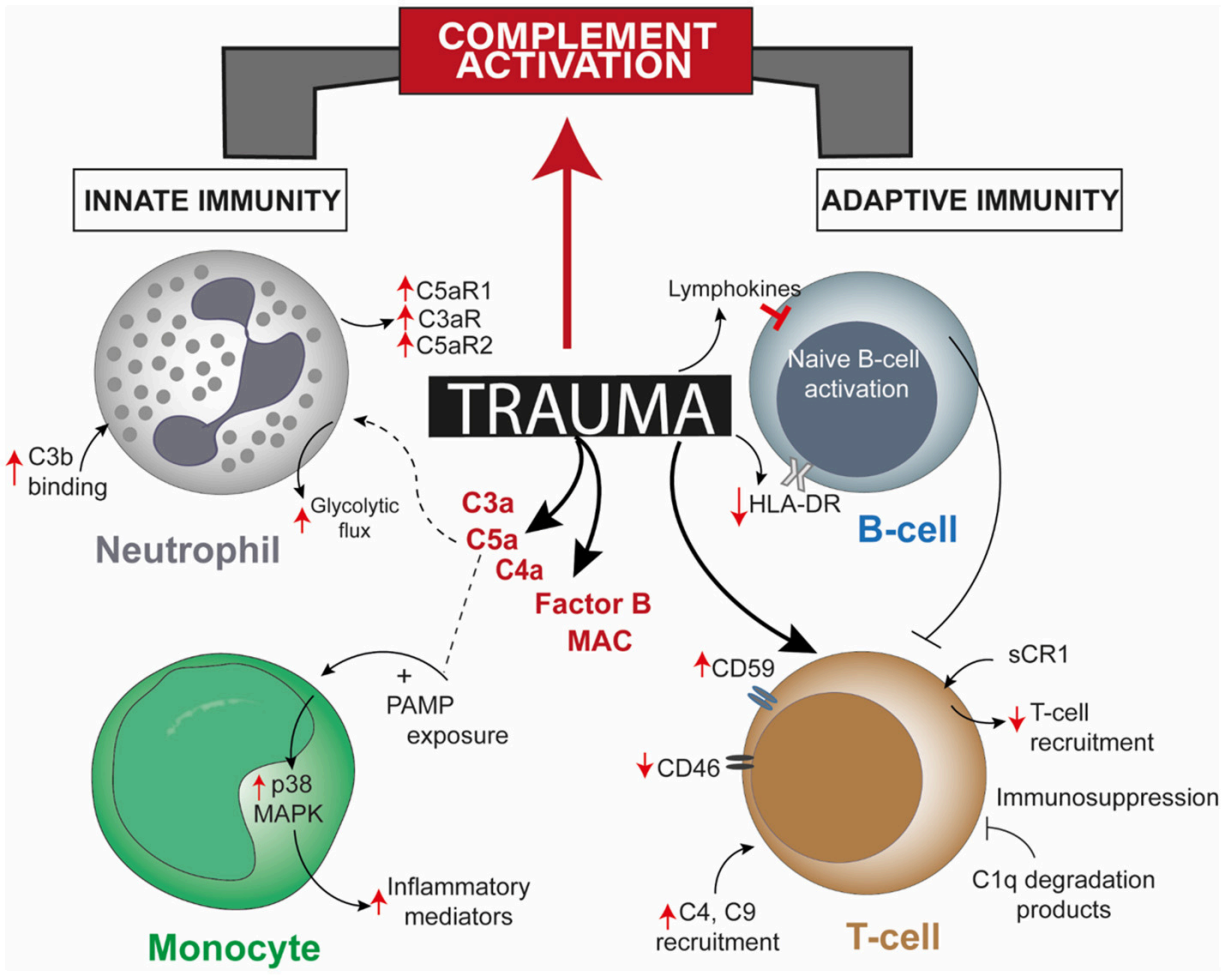
Overview of post-trauma complement activation bridging altered cellular responses of the innate and the adaptive immune system. Trauma alters one or more cellular responses of neutrophils, monocytes, and B-cells and by directly modulating complement regulatory proteins on T-cells, apart from robust activation of complement factors. Though there is a direct effect of trauma on T-cells and B-cells, involvement of mediating complement factors are unreported. CR1, complement receptor 1; HLA-DR, human leukocyte antigen—antigen D Related; MAPK, mitogen-activated protein kinase; PAMP, pathogen-associated molecular pattern.

While the complement system itself maybe dysregulated in the post-traumatic pathophysiology, activation of various other serine proteases, including proteases of apoptotic systems, like granzyme B and cathepsin D, may intensify the extent of complement activity. Damaged cells can also activate factor VII-activating proteases which, together with other proteases, rapidly cleave the complement factors C3 and C5 and generate the respective anaphylatoxins C3a and C5a (Figure 1) (16, 20). In addition to the autonomous action of the complement system after trauma, this ancient fluid-phase innate immune system is intimately associated with the cellular immune responses (13, 21–23). C5a functions not only as an effective chemoattractant for neutrophils, but also upregulates various adhesion molecules on the endothelium, evoking the classical signs of inflammation. C5a exposure leads to various acute defensive functions in neutrophils, including enhancement of phagocytic activity, mounting of an oxidative burst, further inflammatory mediator release and generation of neutrophil extracellular traps. On the contrary, after severe trauma or during prolonged systemic inflammatory conditions that promote complement activation, C5a may become “too much of a good thing” (24, 25). C5a-activated neutrophils have been shown to induce endothelial and mesothelial autologous tissue destruction, for example, of the human omentum, which performs several basic innate and adaptive immune functions (26). Recent studies have indicated that C5a also results in a profound morphological polarization of neutrophils and immunometabolic changes by enhancement of the glycolytic flux (27, 28). C5a was able to generate an alkalotic intracellular milieu in neutrophils and a local lactic acidosis microenvironment despite the absence of an ischemic condition or oxygen debt (27). Yet, it remains unclear to what extent these effects may contribute to known metabolic changes and acidosis after severe trauma. By contrast, deficiency in complement-related factors have also been reported in trauma. Blood neutrophils from trauma patients, which appeared unresponsive to C5a, displayed an impaired oxidative burst activity, less C5aR expression, increased C3b binding activity, and clinically more (infectious) complications (Figure 1) (29). Chemotactic desensitization of neutrophils to C5a was observed in patients with multiple trauma, burn injury or sepsis, indicating dysfunctional danger associated molecular pattern (DAMP) sensing by neutrophils upon excessive complement exposure (29, 30). In accordance with this, loss of C5aR1, C5aR2, and C3aR on neutrophils after multiple injury was found clinically and was correlated to infectious complications and multiple organ dysfunction (31–33). An integrated clinico-transcriptomic approach investigating RNA from leukocytes revealed that lower C5-expression on day 1 after polytrauma was associated with the development of nosocomial infections (34). Monocytes and macrophages are also major players in the orchestrated post-traumatic immune response and are highly responsive to complement factors. C5a is capable of priming peripheral blood mononuclear cells via p38 mitogen-activated protein kinase (MAPK) pathway activation (Figure 1), which in turn results in a significantly enhanced inflammatory mediator release upon secondary exposure to PAMPs (35). In previous studies, the migratory responsiveness of monocytes to C5a but not to fMLP had been found to be suppressed, with a maximum depression from 5 to 7 days after major injury (36, 37). The authors proposed these findings as an explanation for the predisposition of trauma patients to infection and poor wound healing.

When the “first line of defense” represented by physiological barriers, including the skin, is injured, the “second line of defense,” represented by innate immune cells and the complement system is challenged. Proteomic analyses of wound fluids after (surgical) trauma revealed the presence of key complement components including C3 and factor B, in addition to a discrete proteomic leukocyte signature, indicating that as part of the second line of defense, both leukocytes and complement implement the clearance of wound debris and regenerative processes (38). Currently, the role of complement in regard to the “third line of defense,” as formed by the adaptive immune system, is less well known, more so in the context of trauma.

## Trauma: where do complement and adaptive immunity meet?

The complement system and the adaptive immune responses after trauma have been described separately in various literature. Nonetheless, post-traumatic complement activation and its effects on lymphocyte phenotype and function remain elusive. At present, few studies mainly involving trauma injury like traumatic brain injury (TBI), burn injury, and splenectomy after blunt abdominal trauma, have brought the complement system and the adaptive immunity on to a common platform. Injury to the central and peripheral nervous system like TBI, peripheral nerve injury and spinal cord injury (SCI) have offered suitable opportunities to partially understand the basis of complement—adaptive immunity cross-talk. In a murine SCI model, IgM was proven to drive not only the recognition of neoepitopes on damaged cells, but also facilitated on-site complement activation and C3d deposition correlating with injury severity (39). This relation of complement activation to the injury was further bolstered through multiple studies, like, controlled brain contusion performed on strain-specific rats was found to have significant local C3 expression and high T-cell infiltration 3 days thereafter (40, 41). Implicating that mature T- and B-cells could be a pre-requisite for the generation of activated complement factors, Rag1(-/-) mice used to perform a closed head injury study had significantly decreased C3a levels compared to wild-type (WT) mice with no intergroup difference in injury severity (42). Thus, due to this experiential dependence of injury severity on complement activation, healing could also be achieved with complement inhibition. In post reperfused stroke it was seen that treatment with C3aR antagonist diminished infiltration of C3aR-expressing T-cells at the ischemic site, helping in neurogenesis (43). Pain responses due to peripheral nerve injury induced by partial ligation of the rat sciatic nerve could also be attenuated by intraperitoneal injection of soluble sCR1, with diminished macrophage and T-cell recruitment at the injury site (Figure 1) (44). However, the scope of these studies was limited as they did not investigate the cause/s of the observed effects.

Blunt abdominal injury is another example of trauma injury where patients frequently undergo splenectomy. The function of the complement system in attuning adaptive immune responses in the spleen has been underscored by two studies where the role of dendritic cells (DCs) and B-cell responses due to complement factors or receptors in the splenic marginal zone (MZ) has been described. The uptake and transfer of self-antigens by DCs to secondary lymphoid organs serves the crucial purpose of tolerance induction. However, proven through *in vitro* studies, CD8α^+^ DCs resident in splenic MZ can also actively uptake and internalize circulating iC3b opsonized apoptotic leukocytes and these cells require complement receptor (CR) 3 and not CR4 for this purpose (45). Coming to B-cell mediated responses, C4 deficient mice had higher immune complex localization in the splenic MZ and impaired antibody response and class-switching, which was restored when antigen was directed to the splenic MZ (46). Hence, complement seemed to play a role in modulating self-antigen localization such that peripheral B-cell tolerance is maintained. Splenectomy post-trauma affected immune function in terms of reduced T-cell response to phytohemagglutinin, decreased number of lymphocytes, decreased IgM levels, and no changes in C3, C4, and C5 levels (47). Contradicting this former study, other studies concluded that either serum IgM levels did not vary (48) or there was an increase in B-cell population (49). The commonality among them being the unchanged levels of complement factors, a stark demerit of these conclusions was that activated complement fragments were not measured, rendering one inconclusive as to what exact role the complement system might have had in insinuating the adaptive immune responses. In a later study including polytrauma patients, investigation of complement regulatory surface proteins on lymphocytes from the patients showed significantly high CD59 expression 120 and 240 h post trauma and significantly decreased CD46 expression up to 48 h after trauma, with or without splenectomy (Figure 1) (31). Whether an impairment or augmentation of lymphocyte activity is a cause of deregulated complement activation post-trauma was confirmed from burn injury studies, like, generation of C1q degradation peptides in burn patients having an immunosuppressive effect on lymphocytes (50). Serum obtained from major burn injury patients, when subjected to complement inactivating temperatures *in vitro* could affect mitogen-associated lymphocyte blastogenesis, establishing the fact that complement is putatively necessary for lymphocyte development in a trauma event (51). Extending on this concept, in a pig burn wound model (described as 8 burn wounds inflicted for 20 s with a 170°C heated copper rod over a 4 × 4 cm area on two flanks), systemic C3 increased significantly from day 9 and up to 60 days post injury, C4 increment was delayed after burn and a concomitant increase in T-cell infiltration at the wound site was seen on day 3 which declined 21 days post burn injury (52). Additionally, a local increase in C3 and C4 was observed 9 and 4 days post burn respectively, though both decreased after 21 and 9 days, respectively.

As evident from the paucity of relevant studies is that the causality of the complement—adaptive immunity interaction after trauma is still missing. In the following discussion, we focus on aspects of adaptive immunity, from antigen presentation to T- and B-cell functions, which have been proven to be under complement-mediated regulation and vice versa, and how such mechanisms are of importance in the “traumatic” context.

## Complement system and antigen presentation

Antigen presentation is the first and foremost step in priming lymphocytes for their effector functions. This includes processing of exogenous foreign particles, which are in turn presented by major histocompatibility complex (MHC) class II to CD4+ T-cells and endogenous foreign particles are presented by MHC class I to CD8+ T-cells. MHC class II molecules are principally expressed on professional antigen presenting cells (APCs) e.g., macrophages, DCs and B-cells, while all nucleated cells express MHC class I on their surface. However, in addition to conventional antigen-presentation modes, MHC class I can also cross-present, i.e., exogenous antigens can be presented on MHC class I of professional APCs (53). Reduced antigen-presentation functions were initially reported in post-injury macrophages, further having been frequently described in trauma studies (54, 55). For example, reduced antigen presentation and interleukin (IL)-12 and interferon (IFN)-ɤ production after surgical trauma, a diminished population of HLA-DR+ monocytes early in trauma patients and attenuated IL-15 production by DCs following trauma hemorrhage have been reported (56–58). TBI and its impact could be lessened by targeted inhibition of class II-associated invariant peptide, an essential component in MHC antigen presentation (59). Therefore, antigen processing, presentation and co-stimulation of T-cells could well be under the regulation of activated complement factors, particularly in the event of excessive complement activation which has been observed after trauma. Few studies from the last decade could help one get an idea of the role played by complement factors in the uptake of antigens, self or non-self, through DCs and their effect in generating T-cell responses. The **e**xtensively convoluted pathophysiology of trauma can contribute to cellular apoptosis as seen in the spleen, thymus, and the gut and also in neurons after TBI and SCI (60–63) and requires resolution of unwarranted proinflammatory responses. Conversely, the said physiological mechanism can also yield in an imbalance of the inflammatory responses so generated. Suppression of proinflammatory response was identified to be mediated by DCs when a study found C1q and mannose binding lectin as receptors aiding apoptotic cell uptake by immature DCs (iDCs) (64). In connection to this, apoptotic cell removal by microglial cells, which are central nervous system tissue resident DCs, brings about inhibition of IL-6, IL-1α, TNF-α, and IL-1β as opsonization with C1q targets apoptotic cells to DC (65). Another study described that C1q opsonized apoptotic cell uptake by iDCs reduced their surface expression of CD86 costimulatory molecule along with diminished subsequent Th1 and Th17 cell proliferation, generating a contracted inflammatory response (66). This may explain the observed delayed development of immune paralysis in trauma patients, giving rise to poor prognosis as well as the susceptibility to sepsis (67). In a human study, severely injured patients have demonstrated monocyte deactivation, decreased HLA-DR expression on B-cells and dramatically influenced T-cell activation (68). Furthermore, a study showed increase in splenic T-cell and decrease in splenic B-cell 26S proteasome activity concomitant with changes in NF-κB expression after trauma-hemorrhage, suggesting the essential involvement of proteasomes in the regulation of signal transduction in splenic T- and B- cells (69). However, this study did not further describe if the antigen presentation machinery in B-cells is affected anyhow, as proteasome degradation is a key step in cytosolic antigen processing pathway of antigen presenting cells (53).

A complement-enriched environment is necessary for antigen presentation and T-cell differentiation, which is strongly dependent on the type of complement activation products generated (70, 71). Self-antigen presentation after degradation of apoptotic cells is guided by activated C3, contributing to MHC class II antigen processing and presentation (72). In several studies, the role of CR1 and 2 in prolonged antigen presentation have been described for antigen-specific B-cells and macrophage-mediated antigen presentation to T-cells (73–77). Studies have also attempted to describe the mechanistic pathway of antigen-loading efficiency of tetanus toxin-opsonized by C3b and C4b particles (78–80), though the type of antigen spoken hereof is of pathogenic source and may not necessarily explain mechanisms applicable in the context of trauma. Yet, the dynamics of C3 fragment mediated opsonization in purportedly regulating antigen processing is an intriguing premise. C3 activation fragments bound to antigen can delay its degradation in the cytosol by evading its fusion with lysosomes and maintain the antigen-loaded internalized cargo within DCs, a process which modulates T-cell responses to self-antigens (72). Additionally, antigens complexed with C3b were shown to form more stable MHC class II complex, modulating the antigen processing pathway in the late endocytic stage, the loading of the antigen onto MHC and the presentation of the antigen to T-cells resulting in T-cell proliferation (72, 78, 79, 81, 82). Furthermore, high intracellular cAMP in C3aR- or C3-deficient APCs impaired antigen presentation (83). While antigen presentation is facilitated by complement activation products, DC maturation could also be inhibited by iC3b, which exerts its effect by increased extracellular signal-regulated kinase 1/2 (ERK 1/2) phosphorylation and reduced p38 MAPK phosphorylation, resulting in decreased IL-12p70 and increased IL-10 (84). Even anaphylatoxin C5a is a potent effector in priming lymphocytes, because modified C5a peptide EP54 (response-selective agonist peptide) was identified as an adjuvant in DC-mediated T-cell priming (85). In addition to activated C3 and C5 products, elucidated effects of another complement factor C1q have been shown to drive DC-mediated functional activation of T-cells and cross-presentation of exogenous antigen to CD8+ T-cells resulting in greater CD8+ T-cell proliferation (86, 87). The gradual momentum gained by these evidences puts forward a need to confirm the same in trauma. While different experimental confirmations have brought forth the importance of complement activation and degradation products on antigen processing and presentation pathways, *in vivo* and *in vitro* analyses could help in further elaborating how high levels of C3 and C5 activation and/or degradation products generated during trauma could affect these downstream mechanisms.

## Complement and adaptive immunity

Accumulating evidences supporting a possible interaction between the complement system and T-cells have been discussed in some reviews extensively (88–91). In this section we will discuss the relevant studies and recent developments with respect to complement—adaptive immunity crosstalk from the perspective of trauma-immunology.

### Anaphylatoxin-regulated Th17 response

Trauma severity and its outcome appear to be associated with elevated levels of the anaphylatoxins C3a, C4a, and C5a as well as other activated factors, including factor B and TCC (7, 92). Therefore, these intermediates could affect the resolution of the trauma response when adaptive immunity is involved. Invariably, there are obvious differences between sterile inflammation (like trauma) and infectious inflammation (like sepsis), which although can show similar effects, may or may not employ identical pathways to the resultant observed effect. Therefore, the origin of the antigens involved to enunciate the immune response is important. These antigens can be foreign (e.g., PAMPs), self yet foreign or DAMPs, or self-antigens. In PAMP-driven sepsis mice, treatment with anti-C5a antibodies displayed significantly reduced IL-17 levels compared to control-treated sepsis mice (93). C5a can also increase IL12+ DC migration from the peritoneal cavity to the periphery and could prime both Th1 and Th17 T-cells (94). Delving deeper into the exact effect of Th17 T-cells it was seen that, elevated level of IL-17 is generally observed in sepsis and this can be abrogated by a Rho-kinase inhibitor designating it as a participating downstream signaling mediator (95). Th17 cells mediating proinflammatory responses in trauma have been already reviewed extensively, and a few recent studies have investigated into their modulation by the complement system like elevated recruitment of the proinflammatory Th17-cell cohort in a trauma setting, whose inhibition proved to be protective and supported healing (66, 96–98). Interestingly, the DAMP-driven trauma and its associated Th17 responses is caught up in various contradictory results, albeit without the clarification of an intervening involvement of the complement system. When Inatsu et al., obtained peripheral blood mononuclear cells (PBMCs) from third degree thermal injury patients to culture and stimulate these PBMCs with *Candida albicans* hyphae *in vitro*, they found that PBMCs from burn patients had impaired Th17 differentiation (99). Notwithstanding this reported impairment in Th17 phenotype acquisition, subsequent murine studies showed in third degree burn model that Th17 cells were highly recruited at the site of wound as early as 3 h (100) and even 7 days post-injury (101). Few clinical and murine studies could support this conclusion as well for example increased circulating population of CD4+ Th17 T-cells 5 days after trauma in patients with an ISS > 20 (102) and greater IL-17 production by splenocytes in mice which were given an acute scald burn injury of 5 s at 95°C (103). Apart from systemic effects, the local response to trauma hemorrhage was found to effectively increase the Treg:Th17 ratio in the mesenteric lymph nodes, highly likely due to the concomitant decrement observed in circulatory CD103+ DCs (104). A recent clinical study where 114 trauma hemorrhagic shock (THS) and 50 control patients were recruited also showed that, THS patients who developed sepsis later on had lower Th17:Treg cell ratio (105). As sepsis is a common development in later time-points post-trauma, the relevance of IL-17 as a prognostic marker (67) and the contextual relevance of complementopathy in regulating this response could be further validated. IL-17 has also been identified to predict organ damage through computational network analysis of systemic inflammatory markers in blunt injury patients and neutralizing IL-17A attenuated organ damage in a trauma/hemorrhage mouse model (106). Therefore, it is a prerequisite to disentangle the gradually building yet convoluted concept of how exactly Th17 responses are under intricate modulation/s following trauma. This could be dependent on the time and type of injury, local to systemic differences and the inherent inconsistencies found in human and murine systems, apart from the proven role of complement in sepsis as opposed to the unexplored one in trauma. Complement factors and its regulatory components could also be one of those modulators in trauma injury and its yet untapped functions is an opportunity for further investigations.

### Other complement-mediated T-cell responses

ɤδ T-cells have been recently associated in a few trauma-related burn injury and fracture studies for example in regulation of fracture healing, high recruitment of ɤδ T-cells at the site of the wound, wound healing through modulation of myeloid cells and higher neutrophil recruitment in the lungs after trauma-hemorrhage (107–109). A recent study demonstrated how mitochondrial DAMPs can directly interact with ɤδ T-cells and induce synthesis of proinflammatory cytokines, including IL-1β and IL-6 (110). Though unavailable in the context of trauma, complement system's role has been elaborated in PAMP-driven systemic inflammatory response like sepsis. ɤδ T-cells obtained from cecal-ligation puncture (CLP) mice and transferred to recipient CLP mice followed by *in vivo* treatment with anti-C5a improved survival rates of the recipients (93). When these T-cells were obtained from anti-C5a pre-treated CLP mice and transferred to recipient CLP littermates, the recipients displayed reduced survival, indicating that the pathogenic role of ɤδ T-cells was modulated by C5a (93). Extending upon the effect of C5a, increased C5a generation was shown to stimulate neutrophil release of histones, which in turn can induce lymphocyte apoptosis (111). This study used WT and C5aR1 knockout (C5aR1-/-) mice to induce CLP-sepsis and stained apoptotic cells in the spleen by TUNEL, whereby WT CLP-sepsis mice had up to 5 times higher apoptotic cells per view field. The complement—lymphocyte connection could be confirmed for other complement factors as well, like, functionally defective Tregs, along with defective DCs and impaired B-cell switching were found in a 2 year old male patient with C3 deficiency (112). Hence, the concomitant over-activation of complement factors and following consumptive effect that trauma has on them could very well impede the functioning of the adaptive immune system. A seldom explored complement regulatory protein in traumatic conditions is the decay acceleration factor (DAF), which exerted a protective effect in a pig hemorrhage model and was seen to be upregulated in human neutrophils early after polytrauma (31, 113). Imitating central nervous system inflammation by injecting myelin oligodendrocyte glycoprotein (35–55) peptide showed that *Daf1* transgenic mice with a higher cell-surface DAF expression displayed an attenuated disease phenotype and reduced antigen-specific Th1 and Th17 responses, further emphasizing on the role of DAF in T-cell-mediated proinflammatory functions (114). Hence, the robust involvement of complement factors in generation of T-cell responses could affect the mechanisms that are modulated downstream and the evidences explaining these potential mechanisms have been a recent addition to our understanding.

### Potential mechanisms in T-cells

Severe tissue trauma and hemorrhagic shock (HS) is often associated with a significant oxygen deficit as reflected by lactate acidosis and a drop in the base excess (13). The decrease in pH can be manifested systemically and be even more pronounced locally. Low pH environments are known to activate key complement factors and thereby generate C3 and C5 activation products (115) which in principle can contribute to the complement activation and modulation of lymphocyte intracellular mechanisms. Metabolic effects of activated complement at lower concentrations functionally modulated CD4+ T-cells in terms of glutamine utilization and enhanced oxidative capacity of T-cells; whereas a higher concentration of complement resulted in cell death by ATP depletion (116). A combination of TCC and immune complexes trigger CD4+ T-cell activation by inducing phosphorylation of ζ-chain, ZAP-70, Syk, Src, and Lck along with the arrangement of the actin cytoskeleton and effector T-cell functions (117, 118). Upon stimulation, T-cells in C3 knock-out mice have also been shown to display attenuated T-bet transcription factor expression, a factor which determines lineage specificity in CD4+ T-cells (71). Nevertheless, the complement—T-cell association is not just restricted to this. In the last few years, the intimate association of complement mediated signals in T-cell phenotype acquisition has been elaborated through a series of important studies. It was seen that, absence of C5aR and C3aR signaling caused CD4+ T-cells to acquire a Treg cell phenotype in the absence of DCs, through induction of TGF-β and downregulation of complement factor expression by T-cells (119). Thus, autocrine complement activity of T-helper cells gained relevance with a following study showing the generation of intracellular C3a to be driven primarily by cathepsin-L protease synthesized by T-cells (120). A subsequent more recent study in this context showed how T-cell effector function results from intracellular metabolic alterations, modulated by intracellular synthesis of complement products and surface-expressed CD46 (121). Intracellular generation of C3 cleavage products and binding of C3b to CD46 drives the upregulation of glucose and amino-acid transporters and ragulator complex protein LAMTOR which affects cell growth (121). Even intracellular activation of C5 and its effect on NLRP3 inflammasome signaling are necessary for Th1 cell IFN-ɤ synthesis (122). Considering these evidences, it would be interesting to see how and where these mechanisms are affected, when the aforementioned downregulation of CD46 occurs on T-cells following trauma (31) and the role of intracellular C5aR1 in CD4+ T-cells thereafter.

### Complement-mediated B-cell responses

Raad et al. has extensively reviewed how autoantibody generation is a common observation after central nervous system trauma (103). The review discusses in depth the origin of such autoantigen synthesis, which may result due to generation of autoreactive T-cells or even B-cell hyperactivation. Here, the role of the complement system in expediting lymphocyte sensitization and their responses to self-antigens generated following profuse tissue damage after trauma is left to be explored in detail. Ischemia-reperfusion (IR) injury is a common consequence of trauma where the role of complement has been confirmed together with humoral immune responses (51, 52). Clonally specific B-cells and the activated classical complement pathway were shown to be involved in injury pathogenesis, with IgM implicated as the key mediator (53). Additionally, the relevance of CR2 has been demonstrated in inducing IgG and IgM antibody production and their concomitant effects in IR injury pathogenesis (54). Inhibition of spleen tyrosine kinase (Syk), a membrane signaling protein found in B- and T-cells, elicited protective effects by reducing C3 and IgM deposition in tissues in a model of mesenteric IR injury with remote lung injury (55). Opposing existing concepts, C3 was subsequently shown not to be involved in IR injury, whereby a study revealed that surface-expressed CR2 on marginal-zone B-cells did not require C3 activation products to generate IR injury-induced antibodies (56). While nonspecific and specific humoral immune responses have been evaluated in burn injury and SCI, additional observations have been made in major trauma models (123–125). A study by Faist et al. evaluated B-cell function in 30 patients following major trauma over a 21-day period (126). Although the number of circulating B-cells in the trauma patients was not decreased following injury, the number of terminally maturing CIg+ B-cells were significantly decreased compared to controls up to 21 days post-trauma. B-cells obtained from the recruited patients in this study were analyzed *in vitro* and synthesis of IgA, IgM, and IgG by these B-cells were significantly reduced on day 1 after trauma. Later, IgG synthesis decreased on day 3 though total IgG levels were supra-normal. IgM synthesis was diminished throughout the study as compared to the controls (126). This post-traumatic IgM deficiency has also been described in other studies, a fact which is supported by the lack of the lymphokine IL-2 found in patients with multiple trauma (127, 128). Additionally, B-cell derived autoantibodies after SCI are co-localized with C1q on injured neurons, though it remains unclear whether complement activation modulates the production of these autoantibodies (50).

The complement system is furthermore involved in the maintenance of homeostasis by clearing debris, apoptotic bodies, and immune complexes (129). It regulates effector functions of natural antibodies, and C3 cleavage products participate in the opsonization and transport of antigen to the B-cell compartments of the secondary lymphoid tissue (130–132). A detailed role of complement regulators has been realized of late, like CR2/CD21 and CR1/CD35 expression by follicular DCs (FDCs), which function to retain the antigen in the lymphoid follicles required for the activation, proliferation, and antibody generation of B-cells (133, 134). CR1/2 and complement C3 support the localization of IgM-immune complexes in the splenic MZ (135), where B-cells concentrate the IgM-immune complexes onto FDCs. Supporting this, decreased MZ B-cells dramatically diminish the amount of IgM-immune complex on FDCs. The localization of antigen on FDCs has been implicated in optimizing the formation of germinal centre, memory B-cell formation, somatic hypermutation, and IgG class switching (135). Furthermore, in the FDCs of the peripheral lymphoid tissues, including the spleen and lymph nodes, CR1 and CR2 assist in autoreactive B-cell clone anergy by binding and presenting the self-antigen to these B-cells (136). This clearly points toward the differential expression of these two antagonistic complement regulators during the development of human B-cells, a phenomenon which may influence the maintenance of peripheral B-cell tolerance. Deficiencies in upstream complement molecules, including C1q, C2, and C4, may result in inefficient clearance, in the absence of which these products become a source of autoantigens for B-cells and a probable source of an autoimmune response (137). In addition to complement receptors, attachment of C4b to self-antigen and localization of these complexes to CD35 on stromal cells within the bone marrow also regulate the selection of potentially autoreactive B-cells (138). B-cell maturation and self-tolerance being the major foreground of the few players from the complement arsenal, B-cell class switching was shown to be regulated by C3, as C3-deficient patients had abnormal variation in IgG isotypes, including high IgG3, low IgG2, and no IgG4 response (139). These isotypes can in turn modulate the type of adaptive immune responses generated, having an overall effect in subsequent inflammatory responses generated in the said individuals. This indicates but does not yet prove how complement imbalance post-trauma could contribute to altered B-cell responses though further studies could help explain the same.

### Complement-mediated B-cell signaling

The antigen receptors on B-cells, similar to T-cells, are present as multiprotein complexes. Binding to their cognate ligand initiates an intracellular signaling cascade involving translocation to the nucleus and changes in gene expression that dictate the response of the lymphocyte. C3d has been extensively described in mediating signaling pathways, thus linking the innate to the adaptive immunity (140). In this regard, CR1 and CR2 are the best characterized complement regulators that mediate complement-dependent B-cell signaling. C3d interacts with both CR2 and the B-cell-receptor (BCR), causing activation and proliferation of B-cells and the production of specific antibodies. Studies have demonstrated that C3d can bind to CR2, which mobilizes the B-cell signaling complex consisting of CR2, CD19, CD81, and leu-13. Studies in mice lacking either CD19 or CR2 revealed that the CD19-CR2 complex is essential for proper immune function. This B-cell signaling complex confers important co-receptor activities to the BCR. The BCR consists of a non-signaling membrane immunoglobulin and a transmembrane immunoglobulin heterodimer Igα and Igβ bearing immunoreceptor tyrosine activation motifs (ITAMs). BCR stimulation activates two classes of tyrosine kinases: Src-family kinase (Src-protein-tyrosine kinase [PTK], including Lyn, Fyn, Blk, or Lck) and Syk kinase (141). Active Src-PTKs facilitate the phosphorylation and consequent activation of Syk. Activated Syk phosphorylates numerous intracellular substrates mediating B-cell activation, including the ITAMs of Igα and Igβ, and various adaptor proteins, including B-cell linker protein [BLNK or SH2-domain containing leukocyte protein of 65 kDa (SLP-65)]; which in turn leads to the recruitment of Bruton's tyrosine kinase (Btk) and phospholipase C-ɤ(PLC-ɤ) (142–146). Subsequent phosphorylation of PLC-ɤ generates IP3. This molecule orchestrates a cascade of events resulting in Ca^2+^ release from intracellular stores and activation and nuclear localization of the nuclear factor of activated T-cells (NFAT) and nuclear factor “kappa-light-chain-enhancer” of activated B-cells (NF-κB) pathways (147–152).

CD19 is the only molecule within the B-cell signaling complex capable of intracellular signaling. It acts as a membrane adaptor molecule by recruiting signaling intermediaries, including Vav and phosphotidylinositol-3 kinase (PI3K), which enhance Ca^2+^ flux, activation of ERK 1/2, and ultimately B-cell proliferation (153). C3d-opsonized Ag can co-ligate BCR with CR2, consequently improving antigen uptake (74, 154). This complex recruits several copies of CR2 and the B-cell signaling complex, upregulating intracellular Ca^2+^ mobilization and in turn decreasing the threshold of B-cell activation (4). In conclusion, C3d-CR2 binding resulting in CR2-BCR co-ligation reduces the threshold for cell activation by 10- to 100-fold (155) BCR-CR2 co-ligation is also able to inhibit first apoptosis signal (Fas)-mediated apoptosis. In this context, co-ligation results in increased expression levels of anti-apoptotic proteins, including Bcl-2 and the cellular FADD-like interleukin-1-ß-converting enzyme inhibitory protein short form. Invariably, the increase in Bcl-2 appears to inhibit Apaf-1-mediated apoptosis (156).

Whereas the role of human CR2 in B-cell activation is relatively well-established, much less is known about the exact function of CR1 (CD35). In contrast to CR2, CR1 has been described to mediate inhibitory signals (157–159). CR1 ligands include aggregated C3 and aggregated C3(H_2_O), which in contrast do not bind to CR2. Furthermore, CR1 binds to the complement components C3b and C4b and has co-factor activities in their cleavage by factor I to iC3b, a substrate for CD21, and iC4b, respectively (160). It was demonstrated that CR1 strongly reduced the intracellular Ca^2+^ level and phosphorylation of cytoplasmic proteins. Importantly, this inhibitory activity also occurs in the presence of the costimulatory cytokines IL-2 and IL-15 (157).

In trauma, impaired cellular function, including B-cell activation and antibody production, has been described. A recent study showed that the absence of CR1 and CR2 was protective toward secondary damages following closed head injury, including reduced IgM deposition at the injury site (161), though it was not sufficient to gather how are the downstream signaling mechanisms regulated to confer that protection. Further studies could attempt at deconstructing this yet unexplored premise.

## Future perspectives

Considerable knowledge regarding interaction of the complement system with the adaptive immune system has accumulated over time. Nevertheless, there is a scope for experimental and clinical studies to provide an insight into the biology of complement and B- and T-cell interactions particularly after tissue trauma. It is well established that complement receptors are involved in B- and T-cell signaling and function. In the context of trauma, the broadly described role of CR2 in B-cell-signaling cascades could be investigated upon. While CR2 mediates activation of B-cell signaling, CR1 has B-cell inhibitory functions. In addition to CR1 and CR2, studies have also revealed a minor expression of CR3 and CR4 on B- and T-lymphocytes (162). Their expression on leukocytes, especially macrophages has important functions in inflammatory conditions, including tissue trauma and infection, where CR3 and CR4 enhance antimicrobial functions by potentiating leukocyte cell adhesion (163), though their function on B- and T-lymphocytes remains undetermined. Because CR3 is expressed by cytotoxic T-cells, it can be speculated that this receptor may play an important role in lymphocyte functions (162). Like complement regulatory proteins, the receptors for the anaphylatoxins (C3aR, C5aR) appear to be crucial for proper cellular functions of monocytes and neutrophils, though their respective roles in adaptive immune cells have not been fully understood. An early study found C3aR on neutrophils, monocytes, and eosinophils, but not on B- lymphocytes, whereas another study found expression of both anaphylatoxins on tonsillar B-cells (164, 165). While a recent study found C3aR expression both at the mRNA and protein levels within the B-cell (166), to date it remains unclear whether they express C5aR (167).

**Figure 2:**
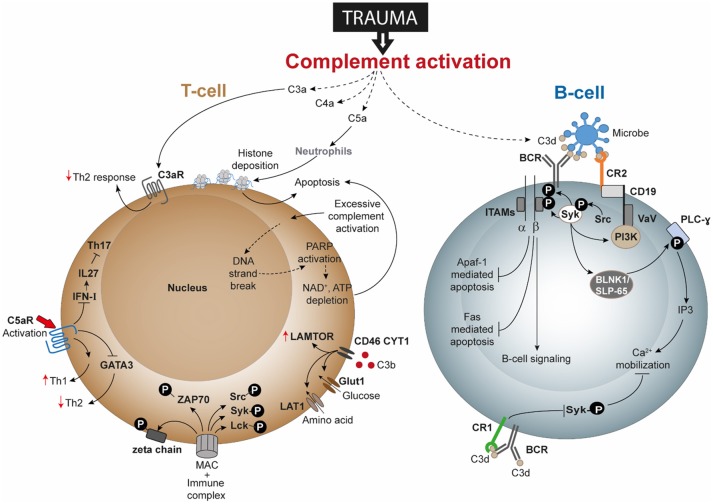
Speculated T-cell and B-cell responses following complement activation after trauma. Activation and dysregulation of complement factors and receptors following trauma may modulate key pathways in T-cells and B-cells. Trauma associated generation of activated complement products have been depicted with dotted lines. All these pathways are discussed in text and are potential areas where trauma research can focus upon. Apaf-1, Apoptotic protease activating factor 1; ATP, adenosine triphosphate; BCR, B-cell receptor; BLNK1/SLP-65, B cell linker protein 1; CD, cluster of differentiation; DNA, deoxyribonucleic acid; FAS: Fas cell surface death receptor; GATA3, GATA3 binding protein; Glut1, Glucose transporter 1; IFN-I, type-I interferon; IL, interleukin; IP3, inositol trisphosphate; ITAMs, immunoreceptor tyrosine-based activation motifs; LAMTOR, lysosomal adaptor and mitogen-activated protein kinase and mammalian target of rapamycin [mTOR] activator/regulator; LAT1, L-type amino acid transporter 1; Lck, Tyrosine-protein kinase Lck; MAC, membrane attack complex; NAD, nicotinamide adenine dinucleotide; PARP, poly (ADP-ribose) polymerase; PI3K, phosphoinositide 3-kinase; PLC-ɤ, phospholipase C- ɤ; Src, Proto-oncogene tyrosine-protein kinase Src; Syk, spleen tyrosine kinase; Th, T helper; VaV, Proto-oncogene vav; ZAP-70, Zeta-chain-associated protein kinase 70.

Several studies have gone on to describe the direct and indirect involvement of the complement system in modulating B-cell responses, albeit not as a consequence of trauma. For example, C3 is required for memory B-cell differentiation and locally generated C5a by peritoneal macrophages is required for chemokine ligand 13 synthesis which is further needed for B1-cell homeostasis (168, 169). Activation of the BCR increased the amount of intracellular C3aR though it could not be detected on the cell surface even after activation. Additionally, B-cell-derived C3 and C3aR appear to have a positive impact on allogeneic stimulation of T-cells (166). Studies of T-lymphocytes have confirmed surface expression of C3aR and C5aR, and revealed that C3aR and C5aR signaling have an important impact on functions of T-cell subpopulations, including Th1 and Th17 responses (170). The effects of trauma on lymphocytes has been barely investigated. Only a limited number of publications indicate that B- and T-cell functions are impaired after trauma, but currently no study exists to clarify the role of the complement system in it. This could indicate that different forms of trauma may affect different pathways of complement and lymphocyte activation, but we do not have any evidence to explain which mechanisms are involved in this putative functional cross-talk. In this context, DAMPs appear to be undermines T-cell survival. Severe injury with consequent tissue damage leads to excessive release of self-DAMPs, including histones (21). Histones released by the effect of C5a on neutrophils have also been described to induce T-cell apoptosis (Figure 2). Therefore, it may be rational to propose that post-traumatic enhanced histone levels is an indirect effect of C5a anaphylatoxin and contribute to T-cell death by apoptosis. Additional investigation is desirable to understand whether post-traumatic C3aR and C5aR signaling is affected. Regarding cells of the innate immune system, C5a can induce C5aR1-mediated morphological changes of neutrophils together with altered chemotactic activity and neutrophils from septic shock patients even displayed decreased C5aR1 expression (28, 171). Activation of C5aR has been described to also enhance proinflammatory Th1 responses and to reduce anti-inflammatory Th2 responses, which should be also addressed in the context of trauma. Because trauma induces a shift toward the Th2 phenotype, it may be speculated that C3aR and C5aR signaling is impaired or their expression is decreased (Figure 2).

With regard to lymphocyte signaling, T-cells have been described to generate intracellular C3 and C5, which in turn can be cleaved and activated and are able to bind to receptors, including C3aR, C5aR, and CD46, thus altering signaling, cell growth, and T-cell responses. What remains unclear is whether autocrine- and/or systemically-generated C3 and C5 cleavage products (e.g., by trauma) may play predominant roles in signaling. It is well established that trauma generates excessive complement activation products. Because complement interferes with receptors on different T-cell subpopulations, including Th1, Th2 and Tregs, it is important to investigate how trauma induces changes to T-cell signaling, function and activity. In this regard, CD46 appears to represent an important receptor in the regulation of T-cell activation and function. For Th1 responses, C3b binding to CD46 and subsequent signaling has also been described to play a critical role in cytokine generation including IFN-ɤ, IL-10, and granzyme-B production in Th1 cells and in their metabolic processes (121, 172). Kolev et al. further established that changes in metabolism influence the resulting CD4+ T-cell functions (i.e., IFN-ɤ and IL-10 production), which may also be influenced in trauma (121). Excessive C3b generation after trauma may alter CD46 function or even expression on T-cell and consequently the functioning of T-cells. Following trauma, the presence of CD46 was found to be decreased on T-lymphocytes (31). Trauma may additionally change the concentrations of intracellular C3 and C5 and their activation, so that an autocrine loop alters CD46 signaling and thus T-cell activation. Further investigations could analyze whether excessive complement activation products after trauma influence T-cell survival, because complement products have been described to induce ATP depletion with subsequent T-cell apoptosis (Figure 2).

Nonetheless, no study to date has provided a mechanistic explanation of a trauma-induced impact on B-cell signaling. One interesting research field may be inhibitory receptors bearing immunoreceptor tyrosine-based inhibitory motifs (ITIMs), which are required for the regulation of B-cell immune responses. To date, numerous inhibitory receptors including CD22 and FcRII have been characterized as possessing one or more ITIMs within their cytoplasmic domain, which generate and transduce inhibitory signals (173). It may be speculated that after trauma, B-cell activation is impaired through a compensatory mechanism to protect the host from further immune reactions mediated by the adaptive system. It could also be possible that in addition to complement interaction with CR1 and CR2, the complement components may interact with these inhibitory receptors on B-cells, reinforcing inhibitory B-cell signaling and thus impairing B-cell activation. Further investigations in relation to the interaction of complement components with other receptors, particularly those with ITIMs could be considered.

Regarding complement therapeutic strategies, several specific and effective approaches have been recently evaluated for their clinical use in inflammatory disorders, including trauma, HS, and sepsis (21). The majority of these investigations focused on the influence of trauma on innate immune responses, including on neutrophil and macrophage function, but less, if at all, on the modulation of adaptive immunity. Therefore, it remains to be investigated to what extent neutrophils, macrophages and also adaptive immune cells contribute to metabolic changes after trauma and whether C5a as a “metabolic switch” may be influenced by specific inhibition (27). C5 cleavage could be inhibited *ex vivo* by small peptide inhibitors, which require future *in vivo* testing for their clinical potential (174). Whereas systemic inflammatory processes after trauma or during infection appear to significantly benefit from central complement blockades, there is no overall benefit for all injured tissues (22, 175, 176). For example, C5aR1 blockade failed to improve uneventful fracture healing, where femoral osteotomy was performed in mice following which immediate and post-12 h, a C5aR antagonist treatment was given (175). Early blockade of C5aR ameliorated both, the IL-6 levels and neutrophil recruitment at the site of injury, but late blockade did not effect in the same. Therefore, the compartmentalized immune response after trauma, involving most likely both innate and adaptive immune reactions, appears to require a specific complement intervention in the future with both adequate spatial and temporal resolution (177). Elaborating on the temporal aspect, the treatment with anti-C5a early post-blunt chest trauma in rats ameliorated subsequent leukocytosis, reduced white blood cell recruitment in the lung, and also decreased trauma induced TNF-α levels (178). As for local effects, 12 h after blunt chest trauma coupled with sepsis induction, C5 deficient mice exhibited decrease in cytokines IL-6 and monocyte chemotactic protein-1 levels in the lung tissue (179). Local healing processes are actively under the regulation of C5a, as its cognate receptor C5aR1 deficiency (C5aR1-/-) affected fracture healing in a mouse model of femoral fracture (180). A remarkable finding in this same study was that, 14 days after fracture in C5aR2 deficient mice, the recruitment of CD8+ T-cells was significantly higher compared to C5aR1-/- mice with fracture, indicating the differences in the elicited function of C5aR1 and C5aR2. Hence, targeted inhibition of complement at the damaged-tissue site is already partly realized. For example, the chimeric CR2-fH construct (mTT30), which can inhibit neuroinflammatory response after traumatic brain injury (181). Similarly, other complement blockers like TCC inhibitors C6 antisense oligonucleotide (OMCI) (182) and CD59-2a-CRIg (183) could turn out to be promising inhibitors and as a result, modulators of the adaptive immune responses. However, in the future, such damage-targeted principles need clinical translation and must not only consider the innate immune response but also the adaptive immune response after trauma, thereby aiming to improve any observed B- and T-cell dysfunction. Another emerging field represents vaccines that influence B- and T-cells in the context of prevention or treatment of infectious morbidity and mortality after traumatic injury, including soft-tissue wounds, human or animal bites, or after splenectomy (184). New effective therapeutics on the C3 level, for example, by compstatin as a small peptide inhibitor and on the C5 level, for example, by NDT9513727 as a non-peptide inverse agonist of C5aR1, need to be tested in the trauma context not only on the innate immune responses but also on the adaptive immune changes (185–187). In the clinic, dysfunctional adaptive immunity after severe tissue trauma requires improved and timely diagnosis. A first approach is represented by clear definition of the persistent inflammation–immunosuppressive catabolism syndrome with its innate and adaptive immune-suppressive characteristics (188). Up until now, the underlying mechanisms remain unclear and need to be elucidated to improve the long-term quality of life of a patient after severe trauma. Overall, complement may represent an innovative therapeutic target in trauma and critically ill patients, because it principally bridges innate and adaptive immunity.

## Author contributions

SC is the first author, EK is the second and MH-L is the Senior author of the paper.

### Conflict of interest statement

The authors declare that the research was conducted in the absence of any commercial or financial relationships that could be construed as a potential conflict of interest.

## Abbreviations

Apaf-1: Apoptotic protease activating factor 1
APC: antigen presenting cell
ARDS: Adult respiratory distress syndrome
ATP: adenosine triphosphate
BCR: B-cell receptor
BLK: B-cell lymphocyte kinase
BLNK: B cell linker protein
Btk: Bruton's tyrosine kinase
cAMP: cyclic adenosine monophosphate
CD: cluster of differentiation
Cig: cytoplasmatic Ig
CLP: cecal ligation and puncture
CR: complement receptor
CR2-fH: Complement Receptor 2- factor H fusion protein
DAF: decay-accelerating factor
DAMP: damage-associated molecular pattern
DC: dendritic cell
DNA, deoxyribonucleic acidEP-54: response-selective molecular agonist peptide
ERK(1/2): extracellular signaling-regulated kinase
Fas: first apoptosis signal
FcRII: Fc gamma receptor
FDC: follicular dendritic cell
fMLP: N-Formyl-L-methionyl-L-leucyl-L-phenylalanine
GLUT1: Glucose transporter 1
HLA-DR: human leukocyte antigen – antigen D Related
Ig: immunoglobulin
IL: interleukin
IP3: inositol trisphosphate
IR: ischemia-reperfusion
ITAM: immunoreceptor tyrosine-based activation motif
ITIM: immunoreceptor tyrosine-based inhibitory motif
LAMTOR: lysosomal adaptor and mitogen-activated protein kinase and mammalian target of rapamycin [mTOR] activator/regulator
LAT1: L-type amino acid transporter 1
Lck: Tyrosine-protein kinase Lck
MAC: membrane attack complex
MAPK: mitogen-activated protein kinase
MHC: major histocompatibility complex
MZ: marginal zone
NAD: nicotinamide adenine dinucleotide
NF-κB: nuclear factor κ-light-chain-enhancer of activated B cells
NLRP3, NACHT: LRR and PYD domains-containing protein 3
PAMP: pathogen-associated molecular pattern
PARP: poly (ADP-ribose) polymerase
PI3K: phosphoinositide 3-kinase
PLC: phospholipase C
PTK: protein-tyrosin-kinase
Rag1: recombination activating gene 1
RNA: ribonucleic acid
sCR1: soluble complement receptor 1
Src: Proto-oncogene tyrosine-protein kinase Src
Syk: spleen tyrosine kinase
TBI: traumatic brain injury
TCC: terminal complement complex
TGF-β: transforming growth factor beta
Th: T helper lymphocytes
Treg: T regulatory lymphocytes
TUNEL: Terminal deoxynucleotidyl transferase dUTP nick end labeling
WT: wild-type
ZAP-70: Zeta-chain-associated protein kinase 70.
